# Evidence of Allomaternal Nursing across One-Male Units in the Yunnan Snub-Nosed Monkey (*Rhinopithecus Bieti)*

**DOI:** 10.1371/journal.pone.0030041

**Published:** 2012-01-11

**Authors:** Baoping Ren, Dayong Li, Paul A. Garber, Ming Li

**Affiliations:** 1 Key Laboratory of Animal Ecology and Conservation Biology, Institute of Zoology, Chinese Academy of Sciences, Beijing, China; 2 College of Life Sciences, China West Normal University, Nanchong, Sichuan, China; 3 Department of Anthropology Program in Ecology and Evolutionary Biology, University of Illinois, Urbana, Illinois, United States of America; University of Delaware, United States of America

## Abstract

**Background:**

Allomaternal nursing, common in several species of social mammals, also has been reported in nonhuman primates. However, the function of this behavior in enhancing infant survivorship remains poorly understood.

**Methodology and Principal Findings:**

The study was conducted on a free-ranging group of the Yunnan snub-nosed monkey (*Rhinopithecus bieti*) in the Baimaxueshan Natural Reserve. Direct observation and *ad libitum* sampling were used to record allocare behavior during a 20 month field study. *R. bieti* exhibits a multilevel social organization in which a large single troop, consisting of over 100 individuals, is divided into many one-male units (OMUs: 6∼41). These OMUs coordinate their daily activities, and feed, forage, travel, and rest together. Here we report on one case of infant temporary adoption in which an adult female from one OMU engaged in allomaternal nursing and cared for an infant from a different OMU of the same troop. This event began when the mother and her five-month-old infant were found to became separated accidentally. The victim infant was observed staying in another OMU. Over the next several days we observed a lactating female in the new OMU to care for and nurse both her infant and the immigrant infant, who also was tolerated by and cared for by the harem male.

**Conclusions and Significance:**

Our findings suggest that lactating primate females are primed to care for young infants and, that the misdirected parental care hypothesis may offer the strongest explanation for allomaternal nursing in *R. bieti*.

## Introduction

Allomaternal care, including allomaternal nursing, defined as a lactating female providing milk for another's offspring [1], is reported to be common in several social mammals such as bats [2], carnivores [3], rodents [4] and seals [5]. In primates, adults of both sexes, juveniles, kin, and nonkin group members are attracted to infants [6]. Although allomaternal nursing is not common among wild primates, it has been reported in approximately 20 species [7] including wedge-capped capuchins (*Cebus olivaceus*) [8], squirrel monkeys (*Saimiri boliviensis*) [9], mantled howlers, *Alouatta palliata*; macaques, *Macaca mulatta*, *M. radiata*, and *M. fuscata*; langurs, *Semnopithecus entellus* and *Presbytis johnii*; gorillas, *Gorilla beringei* [10] and humans [1]. Primate alloparents display a range of caregiving behaviors that include carrying and transporting infants, grooming infants, playing with infants, sharing food with infants, and protecting infants. In the case of tamarin (*Saguinus*) and marmoset (*Callithrix*) monkeys, there is evidence that allomaternal care is critical for infant survivorship [11].

Several theories have been proposed to explain infant attraction and the benefits of allomaternal care in primates. These theories differ depending on the age, sex, and relatedness of helpers. For example under conditions in which limited breeding opportunities result in delayed reproduction, males or females may increase their inclusive fitness by caring for infants that are close relatives [12]. Alternatively, the ‘learning to mother hypotheses’ suggests that juvenile females should provide allomaternal care, if caregiving experience increases the likelihood that they will successfully raise their first offspring [13]. Among primate males, infant care may reflect a form of direct parental investment or used as a mechanism to bond with the infant's mother in order to more easily immigrate into a new social group [11]. Finally, the ‘misdirected parental care’ hypothesis states that social and hormonal factors associated with lactation may result in a mother inadvertently nursing another female's offspring [1]. Roulin argues this could occur in group-living species in which several females give birth during the same period of the year [1].

In this study we present the first evidence of allomaternal nursing in the Yunnan snub-nosed monkey (*Rhinopithecus bieti*). *R. bieti* is an endangered species of Asian leaf-eating primate endemic to high-altitude forests ranging from 3000 to 4400 meters in southwestern China and southeastern Tibet [14]. *R. bieti* live in a multi-level society consisting of 6–41 one-male reproductive units (OMUs) or harems and an associated all-male unit (AMU); OMUs travel, feed, and rest in close spatial proximity throughout the entire year, forming a single large troop [15]. Females typically remain in their natal OMU and do not freely transfer between different OMUs of the same troop [16]. During long periods of the day when the entire troop is resting, infants are observed to move between different OMUs to form temporary play groups (Ren BP. unpublished data). Previous reports of allomaternal behavior in *R. bieti* indicate that adult and juvenile females residing in the same OMU transport, groom, and hold infants [17], whereas adult males principally provide indirect care through tolerance or direct care by protecting infants threatened by predators or other adult males [18]. Intrasexual competition among males for access to fertilizable females is severe [16]. Below, we describe the first documented case of allomaternal nursing and temporary adoption in a wild group of *R. bieti*.

## Methods

**Figure 1:**
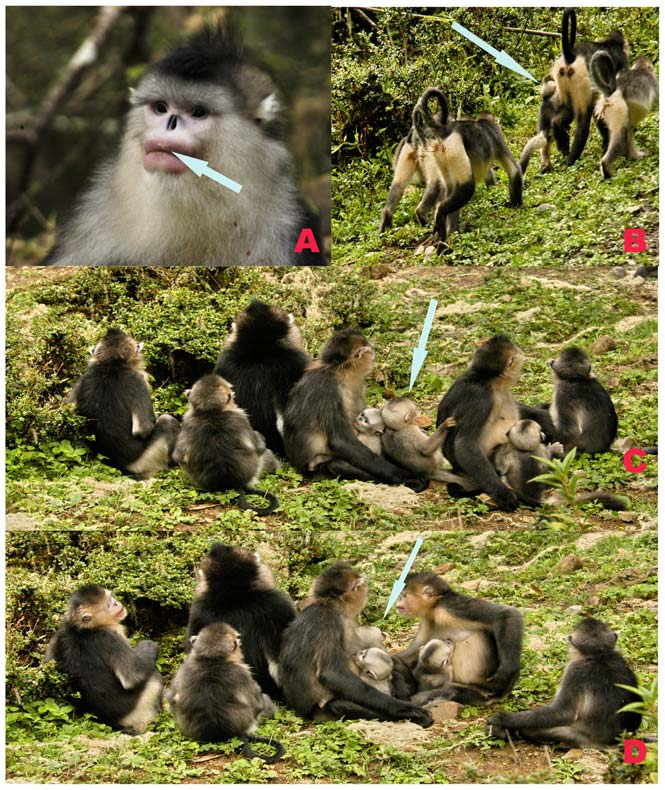
Allomaternal nursing across one-male units in a same group of *Rhinopithecus bieti*. A: Adult male with a white mark on its upper lip. B: The adopted Infant following the OMU. C: The adopted infant residing within the OMU. D: The adopted infant nursing on allomother. (Photograph by Dayong Li).

This study was carried out from May 2008 through December 2009 at Xiangguqing (27°37′N, 99°22′E) in the Baimaxueshan National Nature Reserve in Yunnan, China. The study group contained 95 individuals and eight OMUs, was well habituated to the presence of observers, and was observed daily from a distance of 20–30 meters. If weather permitted, observation would be started from 7:30–8:39 until 17:00–19:00. After the study group was located, only one or two OMUs (3–10 individuals) can be observed each time (3 min.–2 hr.) due to dense jungles and tree canopy at the periphery of the group. Observation ended when the group moved away. Group members could be identified by prominent physical features such as differences in body size, distinctive hair patterns on their head or tail, scars, and pigmentation marks on the face (Fig. 1A). Each resident breeding male marked different OMUs from each other. In our study troop 6 infants were born into four OMUs during the birth season of March through June, 2009. Infant *R. bieti* undergo a series of changes in coat color during their first year of life [19]. Each of the six infants had a different birth date, and therefore we used differences in coat color, physical appearance, and the identity and OMU of the infant's mother to unambiguously recognize each infant. During each observation day we confirmed the presence of each infant in each of the four OMUs.

## Results

On 12 August, at 1705 hr, we observed a non-natal male infant of approximately five months of age residing in the SZ OMU. This OMU consisted of one adult male, two adult females (both lactating), one subadult female, two juveniles, and two infants. The newly arrived immigrant infant played with the two natal infants and sat nearby other group members. Neither the harem male nor any other OMU member directed aggression at the infant. At 1746 hr, the group descended a tall tree and moved onto the ground. The non-natal infant followed, and no SZ OMU member was observed to carry or transport this infant during travel (Fig. 1B). That same day we censused the other three OMUs that had infants and confirmed that the infant in XM's OMU was missing.

We did not track the study group from 13 August to 14 August due to heavy rainfall. On 15 August data collection resumed and at 0932 hr, we observed the non-natal infant foraging on the ground alongside members of the SZ OMU. On the following day, between 1630 hr and 1704 hr, the non-natal infant was observed sitting next to a female who was nursing her offspring. The non-natal infant touched and grabbed the nursing infant while it was being held by its mother (Fig. 1C). At 1648 hr, the non-natal infant was observed suckling one nipple of the lactating female, while her infant was suckling her other nipple (Fig. 1D). When the OMU headed for its nighttime sleeping site (1704 hr), the dominant male transported the non-natal infant a distance of approximately 30 meters while traveling on the ground. On 17 August, we observed the non-natal infant, who appeared to be fully integrated into its adopted OMU. When the OMU began to move at 1733 hr, the non-natal infant initiated contact with the harem male, and held onto his hip as they traveled together a distance of almost 50 meters. The dominant male was highly tolerant, but did not carry the infant during this period of OMU travel. Throughout our observations, no adult or subadult female attempted to carry the non-natal infant as the OMU moved across its home range. And the mother in XM OMU did not retrieve her lost infant.

Beginning 18 August, the non-natal infant was no longer present in the SZ OMU. Instead, the infant was seen traveling with the AMU associated with the study troop. The infant was witnessed more than 10 times in the AMU thereafter. We last observed the infant with the AMU on 21 September 2009. We then observed the infant back in its natal OMU (XM OMU) and with his mother on 13 October. Whether the mother was able to lactate or not was not sure, but the infant was sometimes observed holding mother's nipple in the mouth.

## Discussion

Direct and indirect forms of allomaternal care are widely reported among nonhuman primates [6]. This may reflect the fact that primate life histories are characterized by slow growth and extended infant and juvenile periods. Given the potentially high costs in time and energy of reproduction, the presence of infant caregivers, may enable primate mothers' to redirect investment towards future offspring [11].

In this study, we documented evidence of allomaternal care, especially allomaternal nursing, in a wild troop of Yunnan snub-nosed monkeys. Allomaternal care is common in all species of snub-nosed monkeys [17]. Infants less than 15 days old are carried only by their mothers. By three weeks of age, however, mothers become more permissive and allow other OMU members to care for their infants. During these allomaternal care events, it is the mother that actively retrieves her newborn from the allomother, even if the allomother is of higher rank (Ren BP, Unpublished data). Infant *R. bieti* first begin to feed on leaves and lichen at approximately one month of age. By 4–5 month of age infants exhibit sufficient locomotor skills to travel on their own [20]. At approximately 6 months of age infants obtain most of their food through their own foraging efforts, but may continue to occasionally nurse on their mother for an additional 6–14 months [16]. Although we do not know that exact circumstances that led to a five month old infant transferring from its natal OMU into a new OMU, the non-natal infant became fully integrated into its new social unit and was tolerated and cared for by both the harem male and a lactating female. The harem male transported the infant during travel while the lactating female allowed the infant to nurse along with her own infant. No OMU member behaved aggressively toward the non-natal infant. Our observations of male tolerance and caregiving behavior in *R. bieti* are new compared with patterns of infant caregiving reported in the Sichuan snub-nosed monkey *R. roxellana*. Although *R. roxellana* has been studied for over three decades, only female residents of the same OMU are reported to exhibit allomaternal behavior and assist in transporting another female's infant [16], [21]. Adult male *R. roxellana* have not been observed carrying infants.

Among nonhuman primates allomaternal nursing is not common, but has been reported to occur in several species including two other genera of Asian colobines [7]. And, although kin selection has been offered to explain allomaternal behavior in primates under conditions in which the infants and caretakers are closely related, in species such as Hanuman langurs (*Semnopithecus entellus*) both unrelated and related female helpers are reported to provide care for young [22]. In our observations, we feel that the misdirected parental care hypothesis offers the strongest explanation of allomaternal nursing and temporary infant adoption in *R. bieti*. It is possible that in the case of this lactating female, the secretion of hormones such as oxytocin and prolactin when nursing her own infant facilitated the formation of a temporary bond and nursing tolerance with the lone non-natal infant. We also note, that data collected by Kirkpatrick et al. indicate that infant mortality in *R. bieti* approaches 60% [15]. Therefore, orphan infants are unlikely to survive without allomaternal care and allomaternal nursing.

The circumstances surrounding allomaternal nursing and temporary adoption in *R. bieti* remain unclear and many questions remain unanswered. We do not have information on how this infant became separated from his mother, how it was able to successfully enter the SZ OMU, and how it latter returned to its natal OMU. Given the fact that infants from different OMUs frequently form play groups (Ren, unpublished data), it is possible that during one of these play sessions the infant become separated accidentally from its natal OMU and its mother.

We plan to continue to monitor and study patterns of allocaregiving, female transfer, and adult male-infant relationships in *R. bieti* in order to better determine the set of factors that influence infant mortality and survivorship in this primate species.
